# Diabetes-related foot disorders among adult Ghanaians

**DOI:** 10.1080/2000625X.2018.1511678

**Published:** 2018-09-05

**Authors:** Osei Sarfo-Kantanka, Ishmael Kyei, Jean Claude Mbanya, Micheal Owusu-Ansah

**Affiliations:** a Directorate of Internal Medicine, Komfo Anokye Teaching Hospital, Kumasi, Ghana; b General Surgery Department, Komfo Anokye Teaching Hospital, Kumasi, Ghana; c Department of Medicine, University of Yaoundé 1, Yaounde, Cameroon; d Department of Family Medicine, Komfo Anokye Teaching Hospital, Kumasi, Ghana

**Keywords:** Ghana, incidence, diabetic foot, foot screening, chronic disease

## Abstract

**Background:** Diabetic foot remains a challenge in most low-middle-income countries (LMICs). A severe deficit in data exists on them in sub-Saharan Africa (SSA). Up-to-date data on the longitudinal trajectories and determinants can provide a benchmark for reducing diabetic foot complications in SSA.

**Objective**: The primary objective of this study was to estimate trends in the incidence of diabetic foot and determine predictors in an adult Ghanaian diabetes cohort.

**Design**: The study is a retrospective longitudinal study over a 12 year period.

**Methods**: We applied Poisson regression analysis and Cox proportional hazard models to demographic and clinical information obtained from patients who enrolled in a diabetes specialist clinic in Ghana from 2005 to 2016 to identify longitudinal trends in incidence and predictors of diabetic foot.

**Results**: The study comprised 7383 patients (63.8% female, mean follow-up duration: 8.6 years). The mean incidence of foot disorders was 8.39% (5.27% males and 3.12% females). An increase in the incidence of diabetic foot ranging from 3.25% in 2005 to 12.57% in 2016, *p* < 0.001, was determined. Diabetic foot, with adjusted hazard ratio (HR; 95% confidence interval (CI)), was predicted by disease duration, that is, for every 5-year increase in diabetes duration: 2.56 (1.41–3.06); male gender: 3.51 (1.41–3.06); increased body mass index (BMI), that is, for every 5 kg/m^2^: 3.20 (2.51–7.52); poor glycaemic control, that is, for every percentage increase in HbA1c: 1.11 (1.05–2.25), hypertension: 1.14 (1.12–3.21); nephropathy: 1.15 (1.12–3.21); and previous foot disorders: 3.24 (2.12–7.21).

**Conclusions**: We have found a trend towards an increased incidence of diabetic foot in an outpatient tertiary diabetes setting in Ghana. Systemic and individual-level factors aimed at preventive foot screening as well as vascular risk factor control should be intensified in diabetic patients in Ghana and other LMICs.

**Abbreviations**: BMI: Body Mass Index, BP: Blood Pressure, CI: Confidence Interval, HR: Hazard Ratio, HbA1c: Glycated Hemoglobin, PAD: Peripheral Arterial Disease, NCDs: Non Communicable Disease, SSA: Sub Saharan Africa.

## Introduction

Of the many complications of diabetes, foot disorders represent the commonest, with a lifetime risk of 25% [1]. Diabetic foot defined as the foot of a diabetic patient with ulceration, infection and destruction of the deep tissues, associated with neurological abnormalities and various degrees of peripheral vascular disease (PVD) in the lower limb [1]. It is associated with both short- and long-term increase in morbidity, mortality and lower limb amputation [2,3]. Besides, management involves significant cost inputs regarding investigations and therapy [4]. Among high-income countries, an evident reduction in the incidence of the diabetic foot has been recorded over the past three decades [5–8]. These achievements were mostly through a campaign of increased screening, widespread adoption of multidisciplinary foot clinics and stringent vascular risk factor management [9,10]. On the contrary, a high incidence of diabetic foot, with incidence usually upwards of 10% has been recorded in low-middle-income countries (LMICs), particularly those in sub-Saharan Africa (SSA) [11–14].

In LMICs, particularly those in SSA, health systems are not well equipped to provide care for non-communicable diseases (NCDs) such as diabetes. Instead, they have been set up purposely to combat infectious disease. Many factors including fragmentation of care, limited resource allocation, lack of training among health-care professionals and low health literacy among patients contribute to a high incidence of complication such as diabetic foot [15]. Both community- and hospital-based studies are needed to address this challenge. Such studies will provide the required information on critical local drivers of the high incidence of diabetic foot.

Research on diabetic foot is limited to developed countries, the few conducted in SSA are old and cross-sectional in design. Longitudinal studies detailing foot complications from SSA is non-existent. The primary aim of this study was to estimate recent trends in the incidence of diabetic foot and identify their predictors using an outpatient diabetes cohort from one of Ghana’s leading tertiary hospitals.

## Materials and methods

### Study area and population

The study was approved by the Committee on Human Research Publication and Ethics of the School of Medical Sciences, Kwame Nkrumah University of Science and Technology. This longitudinal retrospective was conducted at the Komfo Anokye Teaching Hospital (KATH), a leading tertiary referral hospital in Ghana. The hospital is located in the second largest city in Ghana, Kumasi. The location of Kumasi in the central point of the transportation network in Ghana makes it accessible to an estimated 10 million people from 6 out of the 10 regions of Ghana and other neighbouring countries. With an estimated prevalence of over 7%, Kumasi has one of the highest urban diabetes prevalence in the country [16].

### The organisation of the Diabetes Clinic

The Diabetes Clinic was set up in 1992 and runs daily clinics receiving referrals from most parts of Ghana with over 22,000 patients enrolled so far. At enrolment, sociodemographic and other relevant clinic information is obtained from patients. They are examined thoroughly for vital signs such as blood pressure (BP) and pulse rates, performed by trained nurses. Each patient rests for at least 5 min before BP measurement while sitting in a chair with both feet flat on the floor, arms supported at the level of the heart on a table. Anthropometric measurement including weight and height was performed by standard methods and body mass index (BMI) derived by dividing the weight in kilograms by the square of the height in metres.

Laboratory investigations including fasting blood glucose, glycated haemoglobin and lipid profiles are recorded. Patients are screened for complications including renal; using urinalysis, creatinine and estimated glomerular filtration rate, eye complications; using an ophthalmoscope, or retinal camera, neuropathy; using microfilament for protective sensation; a 128-mHz tuning fork for the sense of vibration; and ankle reflex testing using a Patella hammer on the Achilles’ tendon. Macrovascular complication was screened for using electrocardiogram, peripheral arterial pulsation and ankle-brachial index testing where indicated. Patients after enrolment are followed up between 2 and 6 times a year based on glycaemic control and complication profile.

### Data collection

**Figure 1.**
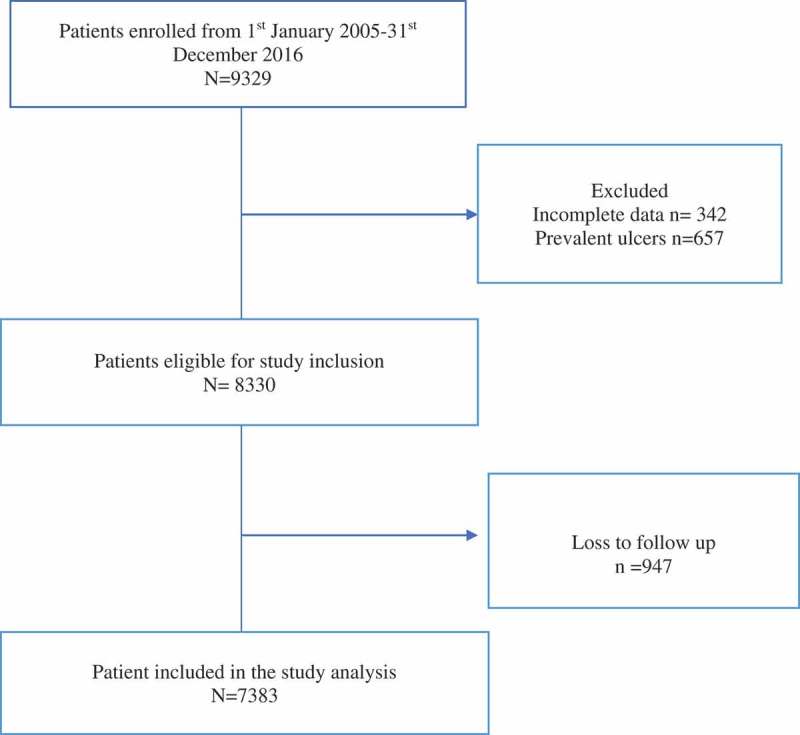
Flow chart of patients enrolled in the diabetes clinic during the study period.

The study involved patients who enrolled in the clinic between 1 January 2005 and 31 December 2016. A flow chart of patient selection for this study is shown in Figure 1. A total of 9383 patients enrolled in the clinic during the period under review. We excluded 342 patients because their records were incomplete, 657 because they presented to the clinic with prevalent ulcers and 947 were omitted because they were lost to follow-up within a year of enrolment. Trained research assistants extracted relevant data from medical records of remaining 7383 patients, after satisfaction with the criteria for diagnosis of diabetes mellitus using the criteria by the World Health Organisation, that is, an elevated fasting plasma glucose level (≥7 mmol/L) on two occasions or oral glucose tolerance test ≥11.1 mmol/L or a self-reported physician diagnosis of diabetes or current use of glucose-lowering drugs [17]. Data recorded include age, gender, duration of diabetes, type of diabetes, hypertension (defined as BP > 140/90 mmHg on two or more readings) [18], BMI, glycated haemoglobin (HbA1c), lipid profile, renal function, current smoking and alcohol consumption.

#### Foot examination

Foot assessment including the presence or otherwise of diabetic foot and the type was recorded. Other examination documented included the presence or otherwise of foot ulcers, pressure marks, callus, amputation/s, posterior tibial or dorsalis pedis arterial pulses and Charcot’s joints. Foot sensation was assessed using a combination of symptoms like paresthesia’s, numbness together with 10-g Semmes Weinstein monofilament examination of the plantar aspects of the great toe, the first and fifth metatarsals and recorded. Inability/reduced ability to perceive the applied pressure at one or more of the three sites with the buckling of the filament was recorded as defective sensation. Vibration sense was tested using a 128-Hz tuning fork using the on-off methods at bony prominences of the leg. Reflexes graded as reduced, normal or exaggerated response were recorded. Peripheral neuropathy was diagnosed based on one or more combinations of sensory, reflex or vibration sense testing abnormalities. Peripheral artery disease (PAD) was diagnosed based on symptoms such as intermittent claudication, leg pain and so on allied to ankle–brachial index measurement (less than 0.9), clinical presentation of ischemia or gangrene. The diagnosis in some instances was confirmed by Doppler ultrasonography/magnetic resonance imaging and angiography.

### Statistical analysis

We defined the primary outcome of the study as the incidence rate of foot disorders, calculated as the number of individuals who reported with a new diabetic foot disorder per person-year under observation. We used a Poisson regression model to examine for trends in the incidence rate of diabetic foot with categorical year variables. We used multivariable Cox proportional hazard models to estimate hazard ratios (HRs; and their 95% confidence interval (CI)) for predictors of diabetic foot during the study period. Covariates shown by previous studies to be associated with diabetic foot disorders were selected for analysis. They included duration of diabetes in years, gender, HbA1c (% increase), type of diabetes, hypertension, nephropathy, statin, aspirin use and previous foot disorders. In bivariate analyses, a *p*-value of 0.10 was set for selection of variables into the final multivariable model with visual inspection for compliance with collinearity assumption. A two-sided *p*-value of <0.05 was considered significant in all statistical analysis with no adjustments made for multiple comparisons. We expressed variables as mean and standard deviation (SD) and dichotomous values as absolute numbers and percentages.

## Results

### Baseline characteristics and sample size

In total, 7383 patients were included in the analysis, as shown in Figure 1. Table 1 summarises the baseline characteristics of the eligible patients. The mean age at diagnosis of the predominantly female (63.8%) cohort was 54.2 ± 11.9 years, mean BMI of 25.7 ± 12.2 kg/m^2^ and mean diabetes duration of 5.5 years. The mean HbA1c was 9.4 ± 2.3, and the mean duration of follow-up was 7.6 years. The total number of person-years of follow-up was 189,541 years.

**Table 1.** Baseline characteristic of eligible patients.

Characteristics	Number (%) 7383 (100%)
Females	4712 (63.8)
Males	2671 (31.2)
Age at diagnosis (years)	54.2 ± 11.9
Type of diabetes	
Type 1	866 (11.7)
Type 2	6412 (86.9)
Other types	105 (1.4)
Body mass index (kg/m^2^)	25.7 ± 12.2
Mean duration of diabetes (years)	5.2
Mean follow-up period (years)	7.6
Smoking (yes)	112 (1.5)
Alcohol (yes)	2654 (35.9)
HbA1c (%)	9.4 ± 2.1
Insulin usage (yes)	1589 (21.5)
Lipid-lowering drugs (yes)	2376 (32.2)
Antihypertensive drugs (yes)	4976 (67.4)
Antiplatelet (yes)	2443 (33.1)

### The incidence rate of diabetic foot disorders

**Figure 2.**
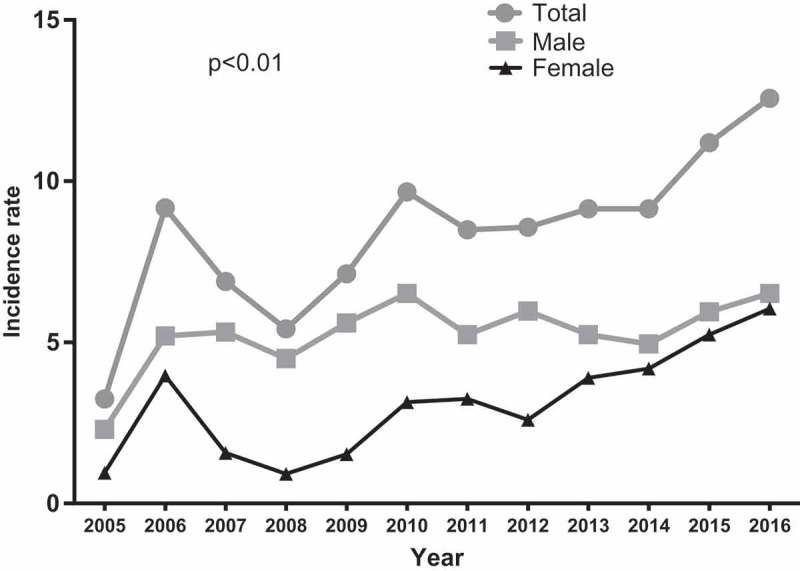
Trends in the incidence rates of diabetic foot disorders in central Ghana.

Figure 2 shows variation in the incidence rate of diabetic foot within the cohort: There was a significant increase in the incidence rate of diabetic foot from 3.25% in 2005 to 12.57% in 2016. The mean incidence rate over the 12-year study period was 8.31%, with the incidence rate among males, 5.06% (2.30–6.52%) higher than in females, 3.33% (0.92–5.24%). The difference in frequency between the two groups varied significantly during the years under study. During the early period under review, that is, between 2005 and 2006, the incidence rate of the diabetic foot was comparable among males and females; however, the period between 2007 and 2012 was characterised by a significantly higher incidence rate in males compared to females. In more recent years, that is, 2013–2016, the gender difference in incidence rate narrowed significantly.

### Foot examination results

Table 2 shows the results of documented foot examination during the study period. Overall 3449 (46.7%) individuals had documented foot examination results. Approximately 66.7% of all individuals examined for foot disorders had one or more abnormalities of the foot. These include 456 (14.6%) with reduced foot pulses/gangrene, 374 (10.2%) with reduced ankle–brachial index recording, 1469 (42.6%) with reduced foot sensation, 779 (22.6%) with callouses, 52 (1.5%) with documented Charcot’s joints, 968 (10.7%) with reduced/absent vibration sense and 179 (5.2%) with amputations.

**Table 2.** Prevalence of risk factors for foot disorders.

Abnormality	At least one component documented *n* (%) *N* = 3449
Absent/reduced pedal pulses	456 (13.2)
Reduced ankle–brachial index	374 (10.8)
Reduced foot sensation	1469 (42.6)
Callus	779 (22.6)
Charcot’s joints	52 (1.5)
Reduced vibration sense	968 (10.7)
Previous amputations	179 (5.2)

### Causes of foot disorders

**Figure 3.**
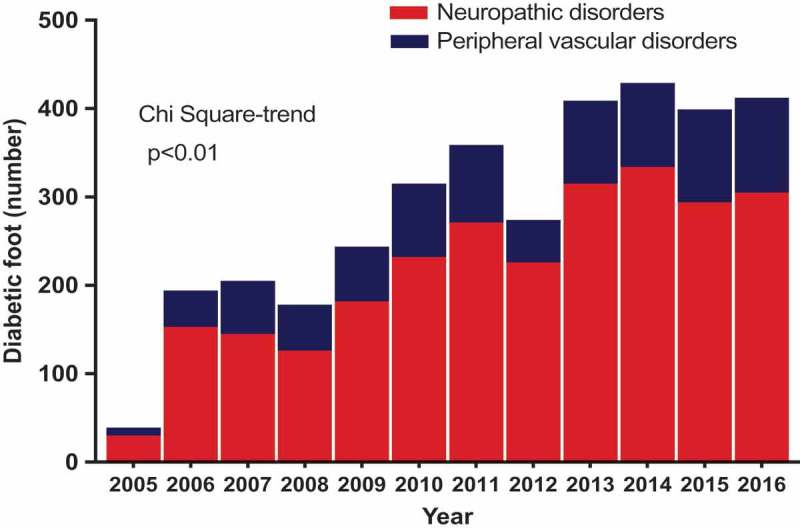
Causes of diabetic foot disorders in central Ghana.

The type of foot disorders presented is shown in Figure 3. In general terms, neuropathic complications including ulcers represented the highest proportion of diabetic foot cases within the cohort representing 74.4% of diabetic foot disorders. PADs/gangrene represented 25.6% of foot disorders, although the percentage of complications attributed to PAD increased over the later years.

### Predictors of foot disorders

Multivariable Cox regression analysis was used to determine predictors of diabetic foot in our diabetes cohort with results shown in Table 3. Duration of diabetes predicted diabetic foot; every 5-year increase in diabetes duration increased the risk of diabetic foot by 2.5-fold (HR: 2.56, 95% CI: 1.41–3.06, *p* = 0.008); male gender increased the risk of foot disorders by 3.5-fold (HR: 3.51, 95% CI: 1.41–3.06, *p* < 0.001); and increased BMI, that is, every 5 kg/m^2^ increase in BMI increased the risk of diabetic foot by about 3-fold (HR: 3.20, 95% CI: 2.51–7.52, *p* < 0.001). A percentage increase in HbA1c increased the risk of diabetic foot by 11% (HR: 1.11, 95% CI: 1.05–2.25, *p* = 0.03), presence of hypertension increased the risk of diabetic foot by 14% (HR: 1.14, 95% CI: 1.12–3.21, *p* < 0.001), nephropathy increased it 2-fold (HR: 2.15, 95% CI: 1.12–3.21, *p* < 0.001) and previous foot disorders increased the risk of foot disorders 3 times (HR: 3.24, 95% CI: 2.12–7.21, *p* < 0.001). Dyslipidemia, lipid and platelet therapy had no predictive effect on the occurrence of diabetic foot in our cohort.

**Table 3.** Multivariate Cox proportional hazard analysis for predictors of foot disorders in the diabetes cohort.

Predictor	Unadjusted HR (95% CI)	*p*-value	Adjusted HR (95% CI)	*p*-value
Gender				
Male	6.93 (3.11–8.00)	<0.001	2.51 (1.88–4.23)	<0.001
Female	1			
Duration of diabetes				
A 5-year increase	3.40 (2.92–3.99)	<0.001	2.36 (1.21–3.16)	0.008
Type of diabetes				
Type 1	1			
Type 2	1.20 (0.8–2.13)	0.17		
Body mass index				
Each 5-kg/m^2^ increase	5.3 (3.11–8.96)	<0.001	3.2 (2.51–7.25)	<0.001
Glycaemic control				
A percentage increase in HbA1c	1.23 (1.08–1.42)	0.003	1.11 (1.05–1.25)	0.03
Dyslipidaemia				
Present	0.84 (0.51–1.36)	0.47	–	–
Absent	1			
Hypertension	2.51 (1.02–1.71)	<0.001	1.14 (1.12–3.21)	<0.001
Nephropathy	5.6 (2.3–8.3)	0.07	2.15(1.92–3.21)	0.002
Lipid therapy	1.02 (0.67–1.32)	0.13	–	–
Antiplatelet therapy	0.56 (0.12–1.12)	0.23	–	–
Previous foot disorders	7.32 (4.31–12.34)	<0.001	3.24 (2.12–7.12)	0.001

CI: confidence interval; HR: hazard ratio.

## Discussion

This study detailing trends in the incidence of diabetic foot in a low-resource diabetes setting represents the first of its kind in SSA, by providing longitudinal data. We have demonstrated that within a 12-year observation period, the incidence of diabetes within the cohort quadrupled. Although in a tertiary referral setting, the majority of patients had no documented foot examination. Additionally, we found an increasing trend towards PVD as an underlying cause of diabetic foot, despite neuropathic disorders of the foot dominating generally. Furthermore, development of diabetic foot in our cohort was predicted by duration of diabetes, male gender, poor glycaemic control, hypertension, nephropathy and previous foot disorders.

In most of SSA and other countries in the LMIC in general, varying incidence of diabetic foot, ranging between 10.2% and 38.9%, has been reported [19–24]. Our study showing a mean diabetic foot incidence of 8.3% is lower than previous findings from LMICs. Perspectives need to be exercised, however, as most of the reported studies on the subject in SSA are old and cross-sectional in design. However, our annual incidence is higher than that observed in developed countries, where a dramatic reduction in the rate of diabetic foot has been recorded over the past two decades [8,25,26]. The North-West and West of Ireland diabetic foot studies conducted in the United Kingdom and the Republic of Ireland reported diabetic foot incidence of 2.2% and 2.6%, respectively [8,27]. Similarly, Muller et al. and de Sonnaville et al. both from the Netherlands indicated an incidence of 2.1% and 1.8%, respectively [28,29]. Ramsey et al. also reported an incidence rate of 2.0% in the United States [30]. It should be noted that both the North-West and West of Ireland studies concentrated on diabetic foot ulcers, the findings, however, pointed to a low incidence of diabetic foot. Diabetic care in Europe and other developed areas is well organised, unlike in LMIC where care is inadequate and at best fragmented, often faced with many barriers [31,32]. Care in developed countries involves the use of multidisciplinary and streamlined pathways as well as monitoring for early symptoms and signs of the diabetic foot complications. These measures have contributed significantly to the reduction of the incidence of diabetic foot. An additional layer of evidence outlining the effectiveness of these measures in reducing diabetic foot can be found in African countries such as Tanzania and Egypt [33,34]. In these countries, the introduction of the Step by Step and the Bridges Projects, involving physician and patient training on early recognition of diabetic foot, as well as multidisciplinary diabetic foot care, has resulted in a significant reduction in the incidence of diabetic foot in both countries. There is increasing prevalence of diabetes in both countries notwithstanding.

Although most foot disorders in SSA are neuropathic in origin, a trend towards an increasing role of PVD has been recently noted [35]. We confirmed this in our study, where despite an increase in the incidence of neuropathic foot disorders in general, there was an increase in the rate of PVD during the later years under study. The increasing role of PVD as a cause of foot disorders can be attributed to the high prevalence of independent vascular risk factors in the general population and most especially among the diabetic population, hyperglycaemia itself being a significant vascular risk factor [36,37]. In our diabetes cohort, for instance, the situation was compounded by the fact that only a third of them were on conventional antiplatelet and lipid-lowering drug therapy. This may have resulted in the increasing incidence of PVD in our patients.

We found a relatively low prevalence of foot examination reported; as less than a half of the patients had one or more documented foot examination during the period under study. This is abysmal compared to the findings from the United Kingdom where a reported 87% of diabetic patients had complete foot examination records [38]. Although a relatively low and somehow comparable prevalence of 56% was obtained in a study in the Netherlands, the study involved general practices, unlike our study which took place in a tertiary referral setting where higher standards are expected [39]. Although an explanation to this finding can be found in the fact that a complete and diligent registration occurs in developed countries for chronic diseases such as diabetes, we cannot discount the fact that foot screening is a critical component of efforts to improve diabetes care [40]. The low prevalence of foot examination records in our cohort can be attributed to a low routine foot examination among diabetic patients, mostly basing the need for foot examination on the presence or otherwise of symptoms and unmistakable evidence of foot abnormalities. It is particularly instructive to note that a significant proportion of our cohort who underwent foot examination recorded a high prevalence of risk factors for diabetic foot as well as complications. We can, therefore, assume that consistent foot screening in this cohort can help reduce the high diabetic foot incidence by enabling earlier, easy to achieve preventive interventions to be employed.

We have shown in this study that diabetic foot in our cohort is independently linked to an increase in diabetes duration, male gender, hypertension, nephropathy as well as previous foot disorders. These findings are consistent with previous results that have identified these factors as being integral to the development of diabetic foot [41,42]. The role of poor glycaemic control in the causation of microvascular complications such as diabetic peripheral neuropathy, for instance, was well established in both Diabetes Complication and Clinical Trial for type 1 diabetic patients and the UK Prospective Diabetes Study for type 2 patients [43,44]. Similarly, the influence of nephropathy and hypertension on the development and advancement of atherosclerotic lesions, especially macrovascular complications such as PVD/gangrene has been well studied [45].

The higher incidence of diabetic foot observed in males compared to females in this study, despite females forming two-thirds of the cohort, brings to the fore the issue of gender-related disparity in health outcomes as a significant public health issue, which require urgent attention [46]. A considerable number of males with chronic diseases such as diabetes, mostly present late for care, do not receive treatment at all or are not compliant with their diabetes treatment. This leads to males suffering severe complications such as diabetic foot at a higher rate than females.

## Study limitations

The strength of this study is in its longitudinal design, a novel, as well as diabetic foot research in SSA, is concerned. It being a retrospective study serves as a limitation because it usually underestimates the incidence rate as individuals tend to underreport and indulge in alternative therapy for such complications. Also, an increased risk of bias may have occurred due to the study involving only a single diabetes centre. Although we have accessed the influence of various factors that might influence the presence of diabetic foot in this cohort, our inability to report on the level of health professional and patient knowledge on diabetic foot as well as other systemic deficiencies also serves as a limitation for this article. Again, future studies on the predictors of foot ulceration in patients with risk factors for diabetic foot will be hugely beneficial.

## Conclusions

To conclude, the incidence rate of diabetic foot quadrupled over a 12-year period in a diabetes cohort in Ghana. Less than half of the group had foot examination records, despite a high prevalence of risk factors for diabetic foot. There is a need to institute patient level and systemic preventive measures for diabetic foot including patient education, task shifting of diabetic foot care, peer support as well as routine screening for symptoms and early remediable signs.
